# Removal of Steel Rust

**Published:** 1879-04

**Authors:** 


Removal of Steel Rust.—According to the *Chemika Zeitung*, articles of steel which have become rusty may become cleansed by brushing with a paste made of 30 parts of cyanide of potassium, 30 parts curd soap, 60 parts precipitated chalk and a sufficiency of water. Great care is required in preparing and using the poisonous mixture.—*Cin. Lancet and Clinic.*

